# Supplementary material for: Complexome profiling of the Chlamydomonas psb28 mutant reveals TEF5 as an early PSII assembly factor
Source: Plant Cell. 2025 Mar 17;37(6):koaf055. doi: 10.1093/plcell/koaf055 (PMC12164590; doi:10.1093/plcell/koaf055)
Supplement: koaf055_Supplementary_Data [file koaf055_supplementary_data.zip › psb28_interactive.zip!/plots/intensity/qualityPlots/peptides/PSAE_Cre10.g420350.t1.2.html]

### Cre10.g420350.t1.2

Trivial name: PSAE  
SeqCoverage [median]: 54.60  
Mass: 10.77 kDa  
  
 

| Sequence | MissedCleavage | Mass [Da] | wtIntensity | muIntensity | Score |
| --- | --- | --- | --- | --- | --- |
| FENQNYAGVTTNNYALDEVVAAK | 0 | 2530.20 | 294686.91 | 368902.26 | 207.43 |
| ILRPESYWFNQVGK | 1 | 1735.90 | 28427.05 | 0.00 | 152.16 |
| VVSVDQSGVR | 0 | 1044.56 | 17776.21 | 5292.48 | 141.48 |
| VVSVDQSGVRYPVVVR | 1 | 1757.98 | 50780.12 | 9878.20 | 105.14 |
| YPVVVR | 0 | 731.43 | 776112.79 | 286512.47 | 150.00 |
| YPVVVRFENQNYAGVTTNNYALDEVVAAK | 1 | 3243.62 | 3280.90 | 0.00 | 53.48 |
|  |  |  | 1171064.0 | 670585.4 |  |
